# Event and Comment

**Published:** 1906-10-15

**Authors:** 


### DENTAL REGISTER.

Vol. LX. October 15, 1906. No. 10.

### EVENT AND COMMENT.

### "Prophylaxis" Defined.

In another place in this issue we publish a criticism of our use of the term "Prophylaxis," by one who deserves the right to criticise, because of his ability as teacher and worker and a writer for more years than we can claim. We are called upon in a most courteous and kindly, but, emphatic manner to give sufficient reasons for an apparent paradoxical if not unjustifiable use of the term "Prophylaxis."

We accept the challenge with hesitation, but no fear, for we know we are called to account by one of our most highly esteemed friends, who aims at the highest good for all, and besides we have a notion that we can show him that we are using the term advisedly and with justifiable authority. We hope, also, that we shall be able to throw some light on this very important method of practice that shall not only enlighten our friend, but other readers of the REGISTER as well, who may be in a similar state of confusion.

We make this preliminary statement with some hesitation for fear we may be accused of an egotistical or irascible disposition, neither of which shall in any way enter into our comment.

In our own words, Dr. Wright defines "Prophylaxis," as something that guards from harm. In a literal sense, and from dictionary authority he is perfectly justifiable in making substantially this form of definition for what he believes we are endeavoring to describe. But he evidently does not believe we have any right to broaden the meaning of this word to include a form of technical treatment which can best be classified under the orginal meaning of the Greek word from which this English term is made. We confess that the appropriateness of the term has had to develop in our own mind with the procedures to which it is applied, until it has now come to have a significance that is distinctive. If we consult the dictionaries we shall find that practically all of them give to the word prophylactic the meaning which Dr. Wright seems to think we are trying to convey. In these same dictionaries there is an apparent effort made at differentiating the meaning of the terms prophylactic and prophylaxis; but it is so weakly done that no real distinction is definitely made except as parts of speech. The words are practically defined as synonomous in meaning. One dictionary defines prophylaxis, as "a preventive treatment for disease," which would surely indicate that it should include therapeutics to some extent at least. In using the term we have perhaps made the error of supposing that practitioners generally had knowledge of the fact that Dr. D. D. Smith, of Philadelphia, had applied this term to his method of restoring the dental organs and tissues when diseased to a normal condition and function. This method of practice is distinctive and differs essentially in method if not in principle from the accepted treatment of certain conditions of disease in the mouth, and involves methods of procedure that could not be classified under the term "prophylactic" without obscuring their technique. But the results of this work are in entire accord with therapeutic principles, though essentially without the use of drugs. Dr. Smith's "Prophylaxis" is not confined alone to polishing the surfaces of the teeth with a stick as Dr. Wright intimates.

Dr Smith in an address delivered before the New Jersey State Dental Society in July, 1904, gave the following definition of prophylaxis as his own conception of what he was trying to do. "Oral prophylaxis is an art or surgical treatment involving manipulative effort, as distinguished from the administration of a systemic medicament or a therapeutic remedy." Again in the same address he says: "The oral prophylaxis treatment, as instituted and recommended by the author, has for its one object and aim the freeing of the oral cavity of conditions of tooth decay, which have become practically universal and the eradication of infection from the same to conserve health and prolong human life." And again in the *Dental Summary* for 1906 he says: "The complete arrest of decay in deciduous teeth and practically of all new decay in adult teeth; a more perfect development of the alveolus; a marked change in color, appearance and structure of the teeth; the return of abnormally sensitive gum tissue to its normal sensibility; the absence of bleeding; the beautiful striation and festooning of the gums, the eradication of all gingivitis and tendency to alveolar pyorrhea; and the extirpation of the mouth infection—the most prolific source of chronic disorder of the stomach, kidneys and lungs, presents to the dental profession matters worthy of investigation, which when fully developed and understood will ally medicine to dentistry in a kinship which can never be annulled."

We cite the above to show that the thought and purpose of what Dr. Smith has been pleased to call "oral prophylaxis" is not only to prevent disease but also to assist nature in restoring to normal conditions tissues already diseased.

Many writers have objected to the Dr. Smith's applying the term "prophylaxis" to his methods, and insist that the term oral hygiene should comprehend this and all other therapeutic measures; and Dr. Wright in his critique apparently endorses Dr. Smith's methods but not his etiologic conclusions. Neither does he seem to give any cures credit to the "prophylaxis" methods.

For some unaccountable reason it seems that it has been almost impossible to bring any large number of dentists to accept the teachings of Dr. Smith as practicable methods. Whether this is because they seem unscientific or impracticable we don't know; but if Dr. Smith with his indomitable persistence has not made the fact clear to all he has undoubtedly converted a few, not only to believe in his methods, but to become enthusiastic practitioners of them, and these all without hesitation will say that they have had more success in treating peridental diseases by this method than by any other therapeutic treatment they have ever employed. We don't believe any fair minded practitioner can look into the mouths of some thirty of Dr. Smith's patients and see the results that have followed a most conscientious practicing of these operative methods and remain unconvinced that therapeutic results have followed surgical interference, and hygienic conditions prevail because of prophylactic conditions produced by "prophylaxis" treatment. And by the prophylaxis method, here referred to, we mean the following out in detail of the mechanical or technical and manipulative methods advocated by Dr. Smith, not only of scaling and polishing, but of operative and prosthetic restorations.

It is here that we hope to throw some light on the apparently discordant use of this term in our essay. When we visited Dr. Smith in Philadelphia and had the good fortune of seeing the results of his efforts in the large number of cases which he presented to us, we gained a new conception of what he was doing, which, as we have before said, compelled our admiration and respect for the man and his methods.

We found that "oral prophylaxis" meant a great deal more than the mere scaling and periodic polishing of the surfaces of individual teeth; in fact other features were so conspicuous that we came near to overlooking the polishing of the surfaces of the teeth in our admiration for the equally important prosthetic restorations that make a continual hygienic or prophylactic effort on the patients part practicable. Of course the teeth were carefully scaled and beautifully polished; but in addition each and every tooth which was in any way impaired, was restored to its normal contour by the most appropiately selected filling materials, used with masterful execution from the technical standpoint; and last, but not least, all missing teeth were supplied, when practicable, by a system of bridgework which served to support the teeth which had been loosened by peridental disease. This bridgework restored normal contour both labially and lingually in a most artistic manner and with materials which would render it easily possible for the patient to keep the mouth in a sanitary condition by the usual mouth toilet.

It is this triple nature of the treatment that broadens and justifies the additional meaning for the term and gives it a distinct meaning that it has not heretofore possessed. And it is this successful attempt at restoring the mutilated and missing teeth to normal contour and function that seems to warrant us in using the term as we have, and attaching to it a meaning that it has not heretofore had.

We do not want it understood that it is practicable by this method to restore all dental arches and gum tissues to their original integrity; but we are willing to say that we have never elsewhere seen such hopeless wrecks restored to entirely comfortable and usable conditions, and to so nearly normal conditions as to assist the patient materially in taking such care of the teeth and gums as to promise a prolonged life of usefulness and freedom from recurrent disease. All this because the patient can get at all the surfaces of the teeth to keep them clean, and besides every tooth is brought into such use in the process of mastication as to insure a high degree of cleanliness because of the excursion of the food over its surfaces. The natural stimulation of the gums and peridental structures because of the uniformly distributed impacts of mastication constitutes also one of the most wholesome agencies for oral health, such as no remedial agency could ever supply. And the polishing of the teeth by the dentist periodically would be only partial if not insignificant in comparison or alone.

It is just this artistic mechanical restoration together with the almost infinite pains that Dr. Smith uses to make his patients teeth clean and cleanable that entitles him to use the term "prophylaxis" to differentiate it from the ordinary methods employed to attain similar results.

It is the completeness of his operations that entitles him to still further extend the term and qualify it under the heading of "oral prophylaxis" because it is his aim to place the entire mouth in a condition of health that will offer no menace to the rest of the body and by so doing to ward off pernicious diseases in remote vital organs. If such results are possible under this form of treatment, could any term be more significantly applied to it than one which has for its derivative meaning "to guard against harm and injury?"

And since it has been necessary to develope a complete system of operative proceedures to accomplish this result, which system is capable of producing definite result when fully applied, are we not justified in appropriating for it a name which more completely embodies its meaning and mission rather than to attach a general term of no specific plan of action, such as oral hygiene, or one signifying merely a degree of effectiveness, such as the adjective prophylactic?

We know of no term that so completely conveys the full meaning of the proceedure and we do not believe the original meaning of the word will be discredited by so great an advance in rescuing the oral cavity from its most troublesome diseases, even though we are not yet ready to believe that a source of disease in other tissues and organs is eradicated.

**Corrections.**

EDITOR OF THE DENTAL REGISTER.

*Dear Sir:*—The August number of the DENTAL REGISTER has just reached me. Permit me to call your attention to three articles therein.

The writer of the first item in "Event and Comment" while not really in error evidently does not appreciate the different conditions under which dental practice is conducted in Great Britain and in the United States. Dental laws in in the United States prohibit the practice of dentistry by anyone who does not possess the required qualification. The dental laws of Great Britain are not aimed at the practice of dentistry by the unqualified, but rather at the deceit of those who claim to be dentists, but have no legal right to that title. The first question considered in all cases of prosecution under the dental act is, did the defendent hold out that he was a dentist? Anyone can practice dentistry all they want to, provided that they can so manage as to avoid passing themselves of as legal practitioners. They cannot collect a bill for work done, but they can collect for material used. In a case reported, one-half of the fee had been paid and the dentist— not on the register, and therefore an illegal practitioner, sued for the balance. There was no complaint of the work. The defense was, that as the dentist was not a legal practitioner, he therefore was debarred from collecting his fee. After a short discussion the magistrate asked the dentist, was the amount paid you for your work, or for the the material, or did it include both? The dentist answered, it was my fee for services rendered, I am now suing for the value of the material used. Judgment was promptly given in the dentist's favor. The law could not be used to assist a dishonest patient.

The case to which you refer was a clear case of malpractice by an ignoramus. He was an illegal practitioner, and yet it is quite possible that, owing to the peculiar construction of the dental act, an attempt to compel him to discontinue practice might fail. In the United States, on the contarary, the dental law is infringed when one not legally qualified practices. It is not what he claims to be, but what he does.

This peculiar and embarrassing defect of the dental act of Great Britain has long been appreciated; it is one reason why quackery is there so rampant. An earnest desire for its removal has been felt for many years, but difficulties we, on this side, cannot fully comprehend, stand in the way. The difficulty is a legal technicallity they have so far been unable to overcome. The profession is not in fault.

The reason why a graduate of an American Dental College is not allowed to practice without other qualification in Great Britain is a point on which there is a general misunderstanding on this side. The withdrawal of legal recognition of all degrees and titles by the General Medical Council of Great Britain, excepting those conferred by its authority or in conformity with the curriculum it established and maintains, when it took charge of the educational concerns of the dental profession in that country, was a wise, indeed, a necessary move. It calls for no apology. It was planned by, and met the approval of wise and good men. It has done a world of good for the profession in Great Britain, and has harmed no one.

Anyone who will take the trouble to look up the course of events that led up to it, and the good which has come from it, will honor the men who brought it about. The idea that it was, or that it now is, a reflection upon American Dental Colleges, or the method of instruction pecular to American Dental Schools, is a misconception. Systematic dental education in Great Britain began the fall of 1799, and on the lines then laid by that excellent teacher, Joseph Fox, it has continued to the present. The curriculum has from time to time expanded to meet the requirements of a growing profession, until it now leaves but little to be desired.

The American dental educational system inaugurated at Baltimore, 1839, was on far different lines. For many years the American system produced by far the best results, and probably does so now. It proved a grand uplift to the dental profession the world over. Under its present auspices dental education in Great Britain has wonderfully developed.

It is a good thing for any country to take the educational affairs of its people into its own hands to foster and protect its own schools and its own industries. It was not a question of merit, it never has been. It was a question of expediency, pure and simple. Let anyone who doubts this look the matter up, from about 1857 to date.

If you will again look up the article in July *Dental Cosmos* referred to on page 424, you will note an error in crediting it to my much esteemed friend, Prof. James Truman. Our names are similiar, but not the same.

There is an error in the last article page 432, that however, is not yours. The oldest dental society in continuous existence is the Pennsylvania Association of Dental Surgeons, organized Dec. 15, 1845. For a few years it met quarterly, but for more than half a century it has met monthly, omitting however, of later years, the mid-summer months as is usual with such societies in the larger cities.

I have been a member of it for over forty years. It celebrated its fiftieth anniversary by a banquet some ten years ago. It has never "shut up shop." It has a membership of about one-hundred. Its last meeting was held on the second Tuesday evening of last June, when it adjounred until the second Tuesday in October, to resume for the fall campaign.

The St. Louis Dental Society will be the third to have a Golden Anniversary. First, The Mississippi Valley Dental Society, 1894, just before it disbanded. Second, The Pennsylvania Association of Dental Surgeons, Dec. 15, 1895.

Kindly note this in your next

Yours truly,

William H. Trueman,

Philadelphia, Pa.

We are glad to make the corrections above noted by Dr. Trueman. We confess that the English ways of dealing with professional men and their affairs have always been a difficult proposition to us, although we have tried in vain to get a right conception of them. The other items were clearly errors of oversight, which we regret.

Harvard Graduates.—We wish also to correct an error in the list of graduates from the dental colleges as published in the September issue. Harvard University Dental School graduated thirty-four this year instead of eleven as we printed it. The eleven graduates should be credited to Howard University.

